# Preoperative Axillary Staging with 3.0-T Breast MRI: Clinical Value of Diffusion Imaging and Apparent Diffusion Coefficient

**DOI:** 10.1371/journal.pone.0122516

**Published:** 2015-03-30

**Authors:** Suvi Rautiainen, Mervi Könönen, Reijo Sironen, Amro Masarwah, Mazen Sudah, Juhana Hakumäki, Ritva Vanninen, Anna Sutela

**Affiliations:** 1 Department of Clinical Radiology, Kuopio University Hospital, Kuopio, Finland; 2 Department of Clinical Pathology, Kuopio University Hospital, Kuopio, Finland; 3 Unit of Radiology, Institute of Clinical Medicine, University of Eastern Finland, Kuopio, Finland; 4 Unit of Pathology and Forensic Medicine, Institute of Clinical Medicine, University of Eastern Finland, Kuopio, Finland; 5 Cancer Center of Eastern Finland, University of Eastern Finland, Kuopio, Finland; University Medical Centre Utrecht, NETHERLANDS

## Abstract

The axillary staging in newly diagnosed breast cancer is under major evolution. The aims of this study were to define the diagnostic performance of 3.0-T diffusion-weighted imaging (DWI) in the detection of axillary metastases in newly diagnosed breast cancer, to assess apparent diffusion coefficients (ADCs) for histopathologically confirmed metastatic lymph nodes in a clinical setting. Altogether 52 consecutive breast cancer patients underwent magnetic resonance imaging and DWI in addition to axillary ultrasound. ADCs of axillary lymph nodes were analysed by two breast radiologists and ultrasound-guided core biopsies were taken. In a separate reading by one radiologist two types of region of interests were used for a smaller group of patients. Altogether 56 axillae (121 lymph nodes) were included in the statistical analysis. Metastatic axillae (51.8%) had significantly lower ADCs (p<0.001). Mean ADCs were 0.663–0.676 x 10^-3^ mm^2^/s for the histologically confirmed metastatic LNs and 1.100–1.225 x 10^-3^ mm^2^/s for the benign. The sensitivity, specificity, and accuracy of DWI were 72.4%, 79.6%, and 75.9%, respectively with threshold ADC 0.812 x 10^-3^ mm^2^/s. Region of interest with information on the minimum value increased the diagnostic performance (area under the curve 0.794 vs. 0.619). Even though ADCs are significantly associated with histopathologically confirmed axillary metastases the diagnostic performance of axillary DWI remains moderate and ultrasound-guided core biopsies or sentinel lymph node biopsies cannot be omitted.

## Introduction

Axillary staging of newly diagnosed breast cancer is under intense research and development. Axillary lymph node dissection (ALND), the former gold standard for axillary staging, has been replaced by less invasive sentinel lymph node biopsy (SLNB) with high accuracy [1,2]. However, many patients still experience the costly and possibly adverse effects of ALND without metastatic lymph nodes (LNs) in addition to the ones removed via SLNB [3]. The importance of axillary staging and ALND in newly diagnosed T1-T2 breast cancers has been debated, and clinical management has been modified in several countries toward an even less invasive approach [4]. This shift has resulted in the need for more accurate imaging tools to diagnose or exclude metastases in designated axillary LNs.

Axillary ultrasound (US) is generally used in preoperative staging of axillary metastases with a sensitivity of ~60% [5,6]. Depending on the biopsy threshold, the sensitivity of axillary core biopsy is 83–89% [7,8]. The reported mean sensitivity and specificity of magnetic resonance (MR) techniques in detecting metastatic LN were high (90%) in a recent review [9].

In clinical practice, breast lesions are frequently evaluated via magnetic resonance imaging (MRI) with dynamic contrast-enhanced and diffusion-weighted sequences. Diffusion-weighted imaging (DWI) exploits the random motion of water molecules, and the contrast depends on tissue cellularity, extracellular tortuosity, and the integrity of cell membranes [10]. Apparent diffusion coefficient (ADC) values are calculated from DWI and displayed as a parametric map. Measurement’s sensitivity to diffusion is adjusted by sequence gradient parameter b-value and DWIs with two or more different b-values are needed for ADC calculations. Evaluation of apparent diffusion coefficients (ADCs) strengthen the discrimination between malignant and benign lesions as it has been shown to exhibit lower ADC values for malignancy [11–14]. DWI and ADC values also facilitate the follow-up of treatment response in neo-adjuvant settings [15]. 3.0-T breast MRI enables higher signal-to-noise ratios, higher spatial resolution, and faster scans than 1.5-T MRI, resulting in improved anatomical details [16] and high diagnostic accuracy when defining malignant breast lesions [17]. Compared to 1.5-T, DWI at 3.0-T is suggested to be more helpful in detecting small cancers (<10 mm) [18]. In breast MRI, the axillary tail area is usually included in the field of view, enabling simultaneous assessment of axillary LNs.

At 3.0-T, improved spatial resolution in axillary LNs in structural sequences may help to avoid areas of fatty hilum when measuring ADCs, while increased visualisation of small malignant lesions improves the sensitivity of DWI. To date, only five studies have evaluated DWI of axillary LNs in breast cancer, none of them solely at 3.0-T [19–23]. At 1.5-T, the reported sensitivity, specificity, and accuracy of DWI vary (84–100%, 77–83.3%, and 80–93.6%, respectively) [19–23] with substantial reproducibility [23]. Previous DWI studies applied a preselected minimal size or other morphological criteria to include LNs. In addition, even though attempts to localise metastatic LNs have been carried out with surgical specimens [21], histopathological references and mean ADC values were previously set with whole ALND material.

The purposes of the current investigation were 1) to define the diagnostic performance of 3.0-T DWI in the detection of axillary metastases in newly diagnosed breast cancer in a clinical setting without LN-exclusion criteria, 2) to assess ADCs for individually histopathologically confirmed metastatic LNs, and 3) to evaluate ADC measurements with information of the minimum and maximum values.

## Materials and Methods

### Patients and Study Design

Kuopio university hospital’s institutional ethics committee approval and written informed consent were obtained for this prospective, single-centre study. Consecutive patients with newly diagnosed breast cancer or Breast Imaging-Reporting and Data System (BI-RADS) [24] 5 breast lesions for whom diagnostic breast MRI and DWI with a standard protocol were performed with clinical indications according to the European Society of Breast Cancer Specialists working group [25] were evaluated as candidates for the present study in our tertiary care institute between April 2011 and December 2012. Primary tumours and suspicious axillary LNs were subjected to US-guided core biopsy according to National Institute for Health and Care Excellence (NICE) guidelines [26]. Interventions were performed by breast radiologists with 4–11 years of experience in breast imaging. In total, 52 patients (mean age 54.9 years) with 121 axillary LNs were individually analysed. Patient demographics and tumour characteristics appear in Table 1.

**Table 1:** Patient Demographics and Tumour Characteristics.

Characteristic	N	%
Patients	52	
Axillae analysed	56	
Bilateral findings	4	
Age in years	54.9	(28–82)^b^
Total Number of Invasive Cancers	54	96.4 [54/56]
Tumour Pathological T Classification		
T1	28	56.0 [28/54]
T2	21	42.0 [21/54]
T3	3	6.0 [3/54]
T4	2	4.0 [2/54]
Tumour N Classification^a^		
N0	23	42.6 [23/54]
N1	12	25.9 [14/54]
N1 (mi)	3	3.7 [2/54]
N2	12	22.2 12/54]
N3	3	5.6 [3/54]
Stage		
1	18	33.3 [18/54]
2	21	38.9 [21/54]
3	14	25.9 [14/54]
4	1	1.9 [1/54]
Tumour histology		
Ductal	41	73.2 [41/54]
Lobular	9	16.1 [9/54]
Tubulolobular	3	5.4 [3/54]
Mucinous	1	1.7 [1/54]
Ductal carcinoma in situ	2	3.6 [2/54]
Grade of invasive cancer		
1	11	20.4 [11/54]
2	29	53.7 [29/54]
3	16	29.6 [16/54]
Total number of LNs analysed	121	
Ipsilateral (lowest)	56	46.3 [56/121]
Ipsilateral (other)	32	26.4 [32/121]
Contralateral	33	27.3 [33/121]
Number of axillae with macrometastases	29	51.8 [29/56]
Size of macrometastases (mm)	14.1	(2–35)^b^
Length LN1 (mm)	16.7	(6–35)^b^
Thickness LN1 (mm)	8.6	(3–21)^b^
Cortical thickness (mm)	4.9	(1–21)^b^

^a^ TNM classification according to American Joint Committee on Cancer.

^b^ Data in parenthesis are range.

### MRI

MR examinations were performed with a 3.0-T scanner (Philips Achieva 3.0T TX, Philips N.V., Eindhoven, The Netherlands) and a 7-element phased-array breast coil. The protocol consisted of five steps. First, T1W-FFE (TR = shortest; TE (in phase) = 2.3 ms; in-plane resolution = 0.48 mm x 0.48mm; 257 slices; slice = 0.7 mm; SENSE factor = 2) was carried out. Second, T2W-TSE (TR = 5000 ms; TE = 120 ms; flip angle = 90 °; in-plane resolution = 0.6 mm x 0.6mm; 85 slices; slice = 2 mm; SENSE factor = 2) was performed. Third, STIR-TSE (TR = 5000 ms; TE = 60 ms; TI 230 ms; in-plane resolution = 1 mm x 1 mm; 90 slices; slice = 2 mm; SENSE factor = 2) was carried out. Fourth, dynamic eTHRIVE (TR = shortest; TE = shortest; FAT suppression SPAIR; dynamic scan time = 58.5 s; in-plane resolution = 0.96 mm x 0.96 mm; 180 slices; slice = 1 mm; SENSE factor = 2.3) was performed with gadoterate meglumine (279.3 mg/ml, 0.1 ml/kg, 3 ml/s) injection. Finally, DWI echo planar imaging (TR = shortest; TE = 95 ms; flip angle = 90 °; FAT suppression SPAIR; in-plane resolution = 1.15 mm x 1.15 mm; 30 slices; slice = 4 mm; diffusion gradients in four directions; SENSE factor = 2.3, two averages) was performed with b-values of 0, 200, 400, 600, and 800 s/mm^2^. The registration of DWI occurred offline before calculation of ADCs using 3–5 b-values including 0, 400, 800 s/mm^2^.

### Image Analysis

Two observers (S.R and A.S, with 4 and 11 years of experience in breast radiology) independently evaluated all axillary MRI data; observers were blinded to histopathological information using a Sectra-PACS workstation (IDS7, Version 15.1.20.2, Sectra AB, Linköping, Sweden). ADC values were measured from primary breast tumours and from glandular tissues of both breasts. Morphological features of the LNs, cortical thickness, width, and length were analysed. All LNs were evaluated at MRI without any size or morphological feature exclusion. T1-weighted, T2-weighted, and dynamic contrast-enhanced images were utilised to localise LNs and to position the regions of interest (ROIs) on ADC maps.

ADC values were measured from each DWI-visible LN in both axillae. LNs were defined as the ipsilateral most caudal visible LN in the assumed “sentinel position” (LN1, n = 56), other ipsilateral position (LN2, n = 32), or contralateral position (LN3, n = 33, from patients with unilateral cancer). At least five ROI measurements were performed for each LN. A small round ROI was positioned to cover ~80–90% of the area of the entire LN in cases of missing hilum. In cases with visible fatty hilum on anatomical T1-weighted images, ROIs were placed on the cortical region. The size of the ROI was set as large as possible while avoiding hilar or surrounding fatty areas.

**Fig 1:**
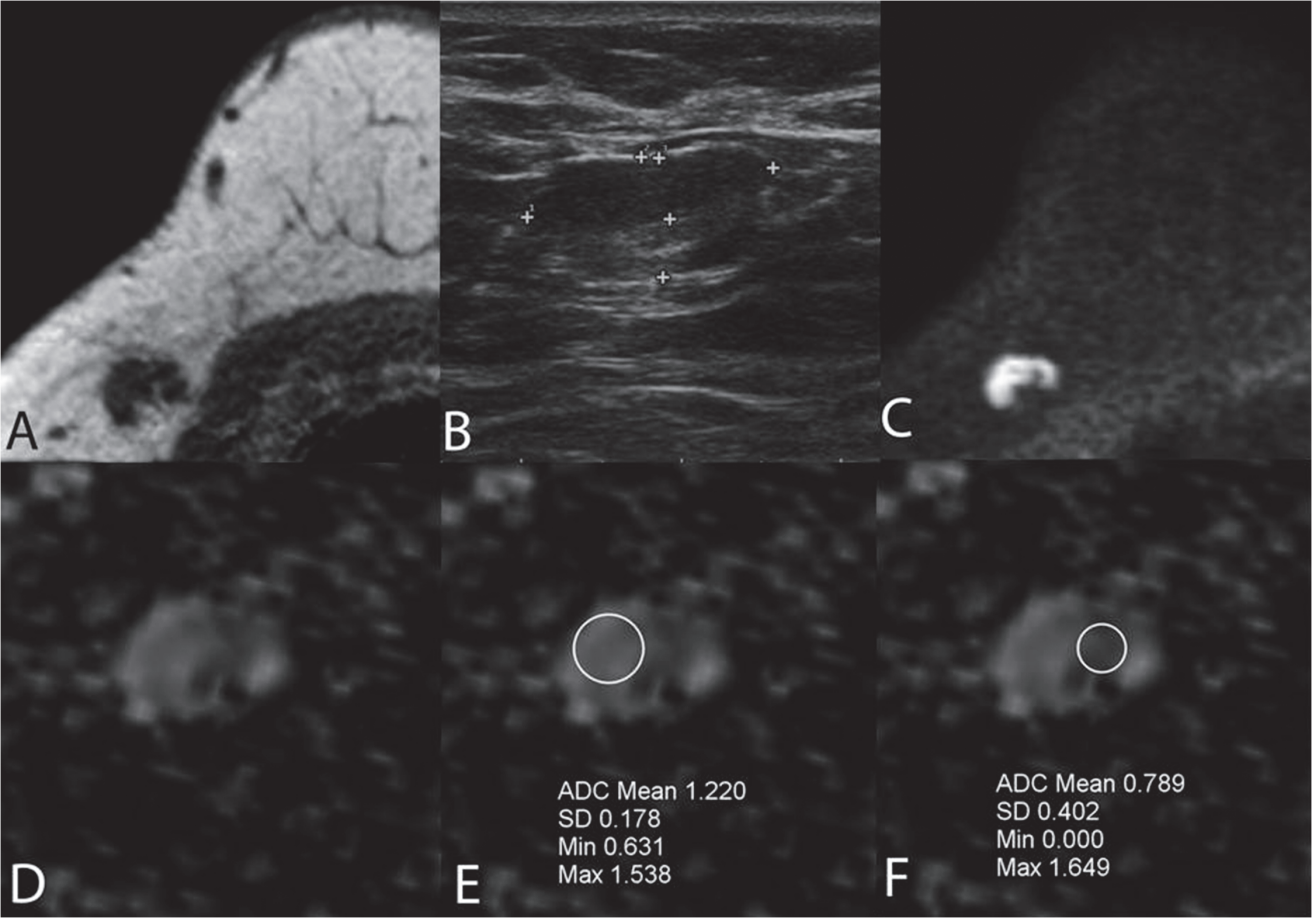
A false positive lymph node due to thickened cortex was true negative on DWI (A-F). Imaging of a 41-year-old female with a BI-RADS 5 lesion in the right breast. An axillary LN with a cortical thickness of 3.9 mm was core biopsied. Final histology revealed a 30 mm, grade 3, T2 ductal carcinoma; the SLNB was benign. No recurrence occurred over a two-year follow-up. The LN was false positive on T1-weighted MRI (A) and US (B) due to thickened cortex, while DWI b = 800 (C,D) was true negative with ADC = 1.24 x 10^–3^ mm^2^/s (cut-off 0.812 x 10^–3^ mm^2^/s). The importance of correct positioning of the ROI and ROI’s information (including minimum value) is illustrated at the area of fatty hilar lobulation (E). False-positive ADCs can be obtained from the cortical area medially if the morphological images are not evaluated or the minimum value of 0 is accepted or is not available at the time of evaluation (F).

A series of 74 LNs (33 randomly chosen patients) was analysed twice by one observer. The first reading employed a ROI with standard information on mean and standard deviation; in a separate session, the second reading included a ROI with information on the minimum and maximum values in addition to the mean ADC and standard deviation. In both sessions, the size of the ROI was made as large as possible while avoiding hilar or surrounding fat, but in the second reading, the ROI was replaced if the minimum value was zero (indicating that the ROI included fat) as illustrated in (Fig 1).

**Fig 2:**
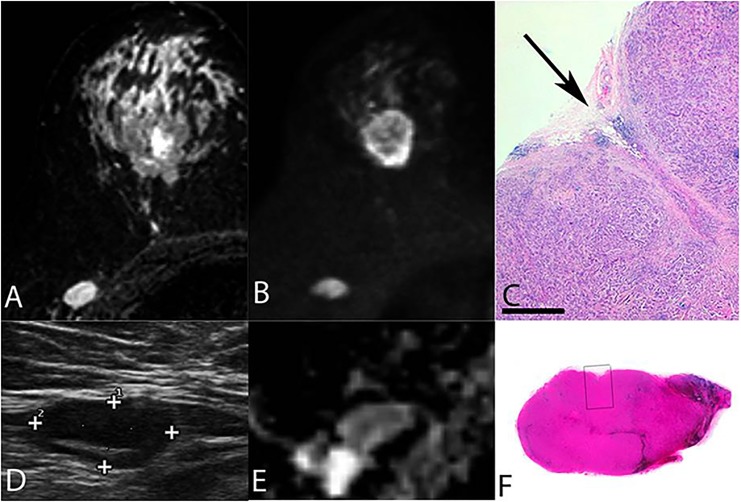
A core biopsied lymph node. Final histopathology indicating true-positive US and DWI findings with metastases (A-F). Imaging of a 62-year-old female with a 31 mm BI-RADS 5 tumour in the right breast. LN that was targeted for core biopsy on axillary US measured 12 x 29 mm with a cortical thickness of 9 mm. STIR, DWI, US and ADC (A,B,D,E) images of this histopathologically confirmed metastatic LN having ADCs of 0.436 x 10^−3^ mm2/s and 0.789 x 10^−3^ mm2/s according to two observers; both of these values were below the threshold of 0.812 x 10^−3^ mm2/s. Histolopatholocigal images (C,F) of core biopsied LN with visual biopsy channel (arrow). Final histopathology indicated true-positive US and DWI findings with metastatic involvement.

All patients underwent axillary US (Esaote MyLabClassC, Genova, Italy, 7–13 MHz linear probe) before or after breast MRI. Lobulated or eccentric cortex, any thickening of cortex >2 mm, and dislocated or absent fatty hilum were considered indications for LN biopsy. Axillary LN biopsies were obtained with 16G core needles aimed at the cortical region of the LN according to a standardized protocol [7]. Each biopsied LN was referenced on MRI, either by comparing the LN’s morphological features and location on US and on MRI or by identifying post-biopsy changes around the LN on MRI in addition to LN morphology (Fig 2).

### Histopathological Analysis

US-guided axillary LN biopsies were placed in 10% formalin and embedded in paraffin after fixation. The samples were cut into 5-μm slices at four different levels and stained with haematoxylin and eosin.

After histopathological confirmation of metastatic disease, patients underwent ALND. If no metastatic disease was detected at this stage, SLNB was performed according to a standardized 2-day protocol including technetium-labelled nanocolloid and Patent Blue dye injection at surgery. Intraoperative frozen sections were analysed according to standard clinical methods. In addition, all palpable suspicious LNs were removed during surgery. When axillary LNs were evacuated, they were subsequently fixed in formalin, embedded in paraffin, and stained with haematoxylin and eosin.

Immunohistochemical staining for low molecular-weight cytokeratins was routinely performed to detect isolated tumour cells or micrometastases. If the SLNB was negative, no further action was taken. The patients were followed for at least 15 months (range 15–39 months) in order to exclude contralateral or recurrent cancer.

### Statistical Analysis

Statistical analysis (SPSS version 19, IBM Corporation, Somers, NY, USA) was performed with the mean ADC values selected from five successive measurements of each LN by each observer. Receiver operating characteristic curves for both observers were used to determine the optimal ADC thresholds for discriminating between benign and metastatic LNs. SLNB-negative and contralateral axillae as well as micrometastases and isolated tumour cells (ITC) were treated as non-metastatic LNs. The area under the receiver operating characteristic curve was calculated to determine the accuracy of ADCs for each observer and the two readings by observer 1. Diagnostic performance (sensitivity, specificity, positive predictive value, negative predictive value, and accuracy) of DWI was calculated with the average ADC cut-off for metastatic LNs from the two observers. Positive and negative predictive values were calculated with Bayes’ formula [27], and 95% confidence intervals were calculated with Wilson’s no-continuity correction formula [28]. Combined sensitivity and specificity values were generated with generalised estimating equations. Statistical comparisons were performed using McNemar’s test or Fisher’s exact test for categorical variables and nonparametric tests for continuous variables. The kappa statistic and Spearman’s correlation coefficient for nonparametric correlations were used to analyse inter-observer agreement. P<0.05 was considered to indicate statistical significance.

## Results

From the 56 axillae ipsilateral to the primary breast tumours (including 4 bilateral cancers) half of the axillae (28/56) underwent axillary dissection; the remaining axillae received SLNB only. According to the final histopathological analysis, 29/56 axillae (51.8%) had macrometastases and 2/56 (3.7%) had micrometastases. SLNB revealed a 3-mm macrometastasis in one patient after a false-negative frozen section, but the patient did not consent to ALND in a second operation. No synchronous or metachronous breast cancers were detected during follow-up.

From the 56 ipsilateral axillae evaluated, 32 (57.1%) core biopsies were obtained, of which 22 (68.8%) were true positive for metastasis, seven (21.9%) were true negative, and three (9.4%) were false negative. After excluding the three core biopsy false-negative cases, 29/32 (90.6%) of the biopsied LNs were reliably identified on MRI with ADC measurements. The majority of metastatic LNs were situated in the lowest possible location in the axillae (LN1, n = 20, 90.9%).

**Fig 3:**
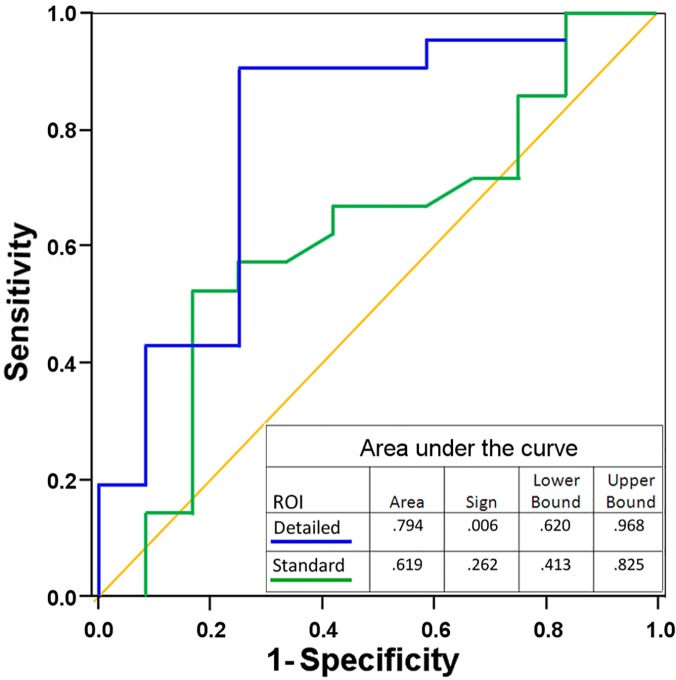
Receiver operating characteristics of readings with two types of ROI information (n = 33). First reading with no information on minimum value in the ROI (standard ROI), second reading with information of the mean, standard deviation, minimum, and maximum values (detailed ROI) improved the accuracy. Diagonal segments result from ties.

The minimum ADC value inside the ROI was helpful when avoiding the fatty areas of small LNs or LNs with thin cortices. In the dataset from observer 1 (33 patients), the ROI with information on the minimum value clearly improved the results compared to the conventional ROI (area under the curve 0.794 vs. 0.619, (Fig 3)).

**Fig 4:**
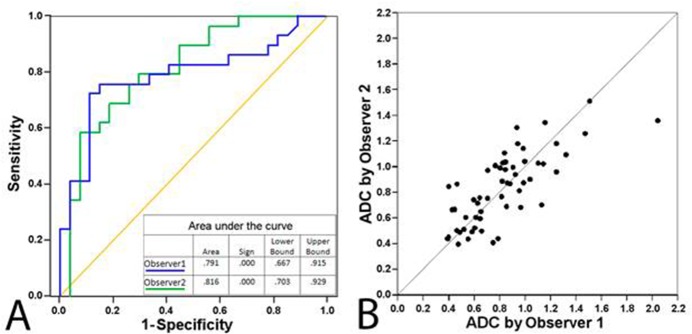
The ADC readings were congruent between observers (A) and (B). Receiver operating characteristics (A) and scatter plot (B) of readings from two observers (n = 56) with ROIs including information of the mean, standard deviation, minimum, and maximum values. Diagonal segments result from ties.

In the entire study population, the mean ADCs for benign and metastatic LNs measured were congruent between observers (ĸ = 0.58, p<0.001; *r* = 0.76, p<0.001, n = 56). The mean ADCs for LN1 with metastases were significantly lower (p<0.001) than ADCs in benign LNs in the ipsilateral axilla for both readers. The ADC cut-off for metastatic LNs was 0.812 x 10^–3^ mm^2^/s. The combined sensitivity, specificity, negative predictive value, positive predictive value, and overall accuracy were 72.4%, 79.6%, 72.9%, 79.2%, and 75.9%, respectively. DWI excluded axillary metastases in 74.8% of the histopathologically negative cases with previously described cut-off value. The diagnostic performance of ADC measurements for both observers is presented in Table 2, and the receiver operating characteristic curves appear in (Fig 4). Furthermore, no statistical difference was observed between ADC measurement values of axillary LNs without previous biopsy in comparison with ADC values of LNs imaged after biopsy (p = 0.268 and p = 0.475).

**Table 2:** Performance Measures of 3.0-T DWI MRI.

	Observer 1	Observer 2	Combined
Sensitivity,%^a^	75.9 (57.9–87.9)^b^	69.0 (50.8–82.7)	72.4 (59.8–82.2)
Specificity, %^a^	81.5 (63.3–91.8)	77.8 (59.2–89.4)	79.6 (67.1–88.2)
Overall accuracy %	78.6 (66.2–87.3)	73.2(60.4–83.0)	75.9 (67.2–82.9)
Positive predictive value^c^	81.5 (61.1–92.5)	76.9 (55.1–90.0)	79.2 (66.5–88.0)
Negative predictive value^c^	75.8 (59.3–87.1)	70.0 (53.7–82.4)	72.9 (60.4–82.5)
Mean ADC for malignant LN x 10^–3^ (mm^2^/s)^d^, range	0.688, 0.392–1.323	0.689, 0.408–1.091	0.689, 0.392–1.323
Mean ADC for benign LN x 10^–3^ (mm^2^/s), range^e^	1.097, 0.476–2.665	1.031, 0.395–1.535	1.064, 0.395–2.665

Performance Measures of 3.0-T DWI MRI in the Differentiation of Axillary Metastases and the ADCs of Axillary LNs with Histopathologic Diagnosis.

^a^Sensitivity and specificity are calculated with an average cut-off of 0.812 x 10^–3^ mm^2^/s.

^b^Data are mean (95% confidence intervals calculated with no-continuity correction Wilson’s formula with a prevalence of 51.8%).

^c^Positive and negative predictive values were calculated with Bayes’ formula.

^d^Malignancy reference on whole axillae in ALND.

^e^SNLB or ALND negative axillary LN with follow-up and contralateral axillary LN.

For the histologically confirmed metastatic LNs (n = 22), the mean ADCs according to observers 1 and 2 were 0.676 x 10^–3^ mm^2^/s and 0.663 x 10^–3^ mm^2^/s, respectively (p = 0.414). The mean ADCs were 1.045 x 10^–3^ mm^2^/s and 1.039 x 10^–3^ mm^2^/s, respectively, for histologically confirmed benign LNs (n = 7) and 1.225 x 10^–3^ mm^2^/s and 1.100 x 10^–3^ mm^2^/s, respectively, for the contralateral LNs (n = 33). From both observers together, five of the histologically confirmed metastatic LNs had ADCs higher (0.842–1.323 x 10^–3^ mm^2^/s) than our cut-off and were therefore false negative. All other 17 cases were true positive. Features of biopsy-confirmed metastatic LNs (n = 22) are summarised in Table 3. When the statistical analysis was performed for all axillae (n = 88), the mean ADCs for metastatic LN were 0.861 x 10^–3^ mm^2^/s and 0.847 x 10^–3^ mm^2^/s (p = 0.712) for observers 1 and 2, respectively. Thus, the histologically confirmed LNs had lower mean ADC values though this did not have statistical significance with both observers (p = 0.002 and p = 0.172).

**Table 3:** Imaging Features and ADC values of Histologically Confirmed Metastatic Axillary LNs.

Patient	Length on US (mm)	Width on US (mm)	Cortical thickness on US (mm)	Cortical thickness on MRI (mm)	Morphology^a^	Location^b^	Observer 1 ADC mean x 10^–3^ (mm^2^/s)	Observer 2 ADC mean x 10^–3^ (mm^2^/s)
1	13.0	7.0	2.4	2.4	2	LN1	0.464	0.502
2	13.0	5.0	3.0	3.8	1	LN1	0.447	0.666
3	28.0	21.0	18.0	21.0	3	LN1	0.392	0.401
4	18.0	10.0	3.1	4.0	2	LN1	0.597	1.011
5	31.0	11.0	2.6	3.0	3	LN1	0.861	0.842
6	35.0	7.0	4.5	7.0	3	LN2	0.966	0.800
7	6.0	5.0	6.0	3.0	3	LN1	0.644	0.759
8	24.0	18.0	9.0	11.0	3	LN1	0.553	0.435
9	16.0	7.0	4.6	4.3	2	LN1	0.520	0.671
10	29.0	12.0	9.0	10.0	3	LN1	0.789	0.436
11	20.0	15.0	8.8	10.0	3	LN1	1.323	1.065
12	26.0	10.0	4.0	3.0	2	LN1	0.655	0.664
13	18.0	9.0	6.6	8.0	3	LN1	0.647	0.591
14	28.0	20.0	9.3	11.0	3	LN1	0.706	0.718
15	22.0	14.0	11.0	14.0	3	LN1	1.144	1.021
16	16.0	9.0	2.3	9.0	3	LN1	0.583	0.492
17	26.0	18.0	9.3	11.0	3	LN1	0.488	0.488
18	28.0	8.0	4.0	4.0	2	LN1	0.399	0.451
19	12.0	8.0	2.6	2.4	1	LN1	0.428	0.650
20	18.0	6.0	3.0	3.0	1	LN1	0.659	0.499
21	15.0	8.0	3.5	3.5	2	LN2	0.749	0.408
22	9.0	4.0	2.5	2.0	1	LN1	1.104	1.025
Mean	19	10	5.9	6.8			0.676	0.663

^a^ Morphology of the analyzed LN;

1 = smooth cortex, 2 = lobulated cortex, 3 = dislocated hilum

^b^ Location in the ipsilateral axilla;

LN1 = lowest visual LN, LN2 = other ipsilateral position

The diagnostic performance figures for DWI to predict high axillary burden (N2-3 disease) were as follows; 70.6% for sensitivity, 62.2% for specificity, 46.2% for PPV and 82.1% for NPV. The corresponding results for axillary US were 82.4%, 51.4%, 43.8% and 86.4% respectively.

From the group of all patients with invasive carcinoma, macrometastases were found in 28 axillae (50.0%). DWI MRI was false negative in 28.3% (8/28) of cases and 4 of these cases had high axillary burden (N2-3). The maximum size of DWI false negative metastasis was 8.0–19.0 mm and the number of positive LNs varied between 4–13 (mean 6.0). Axillary US was false negative in only one of these cases and core biopsy was not obtained. In the remaining 7 cases core biopsy showed metastatic deposits.

In this material, the mean ADC was 0.749 x 10^–3^ mm^2^/s for malignant breast tumours and 1.602 x 10^–3^ mm^2^/s for normal glandular tissue. Table 4 contains comparisons of these values with other published results.

**Table 4:** Comparison of ADC values from Breast Tissue, Malignant Breast Lesions, Metastatic LNs and Diagnostic Performance of DWI.

Reference and year	MR field strength (manufacturer)	Number of patients	B-values (mm^2^/s)	ROI type and placement	ADC normal breast tissue^a^	ADC malignant tumour ^a^	Number of analyzed LNs	ADC mean metastatic LN ^a^	LN ADC cut-off ^a^	Sensitivity (%)	Specificity (%)		Accuracy (%)	
Cakir et al. [11]	3 T (Philips)	52	0, 50, 850, 1000, 1500	round, excluding haemorrha-gic, cystic, and necrotic areas	1.66	0.92								
Park et al. [14]	3 T (Siemens)	110	0, 1000	manually placed, round	N	0.88								
Nogueira et al. [13]	3T (Siemens)	53	50, 200, 400, 600, 800, 1000, 2000, 3000	including the area of highest hyperinten-sity, 0.10 cm^2^	1.99	1.08								
Dong et al. [12]	3 T (GE)	87	0, 800	round, 10–20 mm^2^, excluding haemorrha-gic, cystic, and necrotic areas, mean of three ROIs	1.83	1.19								
Bogner et al. [17]	3 T (Siemens)	51	0, 850	avoiding fatty and necrotic tissue	1.95	0.99								
Scaranelo et al. [23]	1.5 T (Siemens)	74	50, 300, 700, 1000	entire LN or cortical region	N	N	65	0.694–0.706	N	83.9		77.0		80.0
Fornasa et al. [20]	1.5 T (GE)	215	0, 800	round, ≥4–5 mm diameter	N	N	43	0.878	1.09	94.7		91.7		93
He et al. [21]	1.5 T (GE)	136	0, 500, 800	round, most enhanced area (≥20 mm^2^)	N	1.273, b = 500 1.455, b = 800	251	1.369, b = 500 1.182, b = 800	1.680, b = 500 1.351, b = 800	97.0, b = 500 95.8, b = 800		54.4, b = 500 65.8, b = 800		N
Luo et al. [22]	1.5 T (Siemens)	36	0, 800	round, ≥3 measure-ments	N	0.792	79	0.787	0.889	82.2		82.4		82.3
Chung et al. [19]	1.5 T/3 T (Siemens/ Philips)	110	0, 1000	entire LN, ≥3 measure-ments	N	N	110	0.690	0.90	100		83.3		93.6
Present study 2014	3 T (Philips)	67	0, 200, 400, 600, 800	round, ≥5 measure-ments on cortex /entire LN	1.602	0.749	121	0.698	0.812	72.4		85.2		75.9

^a^ x 10^–3^ (mm^2^/s)

## Discussion

The current investigation demonstrates that ADCs can be evaluated even in small axillary LNs as needed in the clinical setting. Furthermore, minimum ADCs from ROI information were valuable in this analysis, especially for LNs with thin cortices to exclude fatty area contaminations. In contrast to the majority of previous studies, we also evaluated ADCs from histologically confirmed metastatic or benign LNs. Previous reports were promising, yet most of them lacked histopathological verification and had selected populations. The previously reported ADC cut-offs for metastatic LNs varied between 0.889 and 1.351 x 10^–3^ mm^2^/s [19–23]. Our results are similar to those from the most recent study [19]. Nevertheless, previous studies are not directly comparable to the current investigation due to different equipment and inclusion criteria.

Very small LNs are thought to be more difficult to measure reliably, and hence previous studies limited the size of the LNs as well as the size of the macrometastases [19–23]. On the other hand, even small LNs can have metastases, and our study indicates that ADC measurements from small nodes are feasible and the higher spatial resolution and signal-to-noise ratio at 3.0-T probably benefit analysis.

DWI of the axillary LNs is associated with several unresolved issues related to ROI quantity, size, and placement, issues that may result in varying measurement outcomes. Furthermore, no consensus exists on imaging technique and appropriate b-values. B-values of 50 and 850 s/mm^2^ on 3.0-T MRI were defined as optimal for breast tumours in a previous study evaluating the diagnostic quality of DWI [17]. However, it is not clear whether the same b-values are optimal for LNs and should be used to determine the threshold values for metastasis. In our study, ROI size was made as large as possible while avoiding hilar or surrounding fatty areas by exploiting the information for the ROI’s minimum value, leading to more precise mean ADCs without possible contamination. This benefit proved to be essential to the accuracy of measuring small LNs and LNs with thin cortices. In the comparative series by observer 1, diagnostic performance was remarkably better when the analysis included information of the minimum ADC of the ROI.

Preoperative staging of the axilla by US and needle biopsies reduces the need for SLNB by 50% in axillary positive patients [29]. On the other hand, comparable outcome results for patients with <3 or no metastatic LNs who fulfilled other inclusion criteria in the ACOSOG Z0011 trial [4] resulted in a less-invasive surgical approach that avoids ALND. This new approach inevitably increases the number of SLNBs in institutes that formerly performed axillary US staging and US-guided biopsies although the relevance of SLNB in clinically and US-negative axillae has recently been questioned [30]. Therefore, any compensatory new procedure should ideally have sufficient sensitivity and specificity relative to the index test. Unfortunately, although DWI in its present form is an intriguing non-invasive test, it does not yet offer dramatic improvements in accuracy.

Ultra-small super-paramagnetic iron oxide-enhanced MRI has been superior to current clinical techniques [9] in detecting axillary involvement in breast cancer. The clinical significance of this method is yet to be determined. Positron emission tomography or positron emission tomography/computed tomography (PET/CT) has mean sensitivity and specificity of 63% and 94%, respectively [31]. Thus, in breast cancer patients, the accuracy of DWI is similar to that of PET/CT, with the benefit of no radiation exposure. DWI data are easily obtained and are readily available in the same session as breast MRI.

Whole-body DWI studies are becoming more utilised in clinical practice. A recent meta-analysis uncovered no significant difference in the detection of primary and metastatic malignancies between whole-body DWI and PET/CT [32]. Adding ADC measurements of LNs and other metastatic foci to the visual analysis of whole-body DWI may have future clinical relevance in the follow-up and evaluation of treatment response. At its current stage DWI and ADC measurements remains unreliable with insufficient accuracy.

Our study has limitations. The number of evaluated LNs remained relatively small (n = 121), though larger than in most previous studies. Additionally, breast tumours are visible on MRI and thus axillary LNs are difficult to evaluate in a truly blinded manner and in addition there are no consensus on used b-values or ROI selection. Further studies are needed to standardize DWI b-values and ROI measurements on ADC maps.

In conclusion, even though ADCs are significantly associated with histopathologically proven axillary metastases evaluated via 3.0-T MRI, the US-guided core biopsies or sentinel lymph node biopsies cannot be omitted based only on DWI. Axillary staging using DWI is not sufficiently accurate to replace SLNB, and although the information on ROI’s minimum value improved results somewhat, the usage of ADC measurements for axillary lymph nodes at this stage does not give enough reliable information to be applicable in clinical use.
