# Correction: Preoperative Axillary Staging with 3.0-T Breast MRI: Clinical Value of Diffusion Imaging and Apparent Diffusion Coefficient

**DOI:** 10.1371/journal.pone.0133111

**Published:** 2015-07-14

**Authors:** The PLOS ONE Staff

## Notice of Republication

This article was republished on June 22, 2015, to correct Figs 1 and 2, which were darker than intended. The publisher apologizes for the error. Please download this article again to view the corrected version.
